# A Rare Case of Progressive Encephalopathy in a Sickle Cell Trait Patient: A Case Report

**DOI:** 10.7759/cureus.45936

**Published:** 2023-09-25

**Authors:** Mohammad A Alzayadneh, Khalid A Alsherbini

**Affiliations:** 1 Neurology, University of Tennessee Health Science Center (UTHSC), Memphis, USA; 2 Neurology/Neurocritical Care, University of Tennessee Health Science Center (UTHSC), Memphis, USA

**Keywords:** progressive encephalopathy, stroke, sickle cell complications, sickle cell trait, cerebral fat embolism syndrome

## Abstract

Fat embolism syndrome (FES) is one of the underdiagnosed and underrecognized complications that can happen in multiple medical and surgical conditions. FES can manifest in a broad spectrum of signs and symptoms and affect multiple organ systems in the human body. One of the most commonly involved is the central nervous system (CNS), mainly the brain, which can be involved in different ways, and the presenting symptoms can vary in type and severity. One of the most common causes of FES is trauma, mainly a long bone fracture or any orthopedic injury. However, one of the rare causes of FES is sickle cell disease (SCD) and thalassemia. Generalized and vague presenting symptoms, the rarity of FES, and the absence of well-defined diagnostic criteria make it a challenging diagnosis for healthcare practitioners. FES diagnosis is usually made after having a high index of suspicion in patients with underlying risk factors that can precipitate and contribute to the pathophysiology of FES. Moreover, the diagnosis is usually reached after excluding other more common and treatable conditions.

## Introduction

Fat embolism syndrome (FES) is a well-documented complication that can arise from long bone fractures and orthopedic surgeries. It is characterized by the emergence of various symptoms, including respiratory failure, neurological issues, skin rashes, and thrombocytopenia [1]. A less-common form of FES, known as atraumatic FES, occurs infrequently and results from extensive bone marrow necrosis (BMN), which can be caused by various factors, including sickle cell disease (SCD) [2]. BMN leads to the release of fat globules and hematopoietic tissue into the venous circulation. When BMN is severe, it releases a significant amount of fat droplets into systemic circulation, giving rise to FES and the characteristic multiorgan dysfunction [3].

FES can potentially affect any system within the human body because of the presence of fat droplets within the peripheral microcirculation [4]. While all patients eventually develop type I respiratory failure, this symptom often does not appear until several hours or even days after initial presentation. A similar delay is observed in the involvement of the nervous system and the development of thrombocytopenia and other hematological and biochemical abnormalities [5].

Alongside the aforementioned causes, there are also rare ones, such as thalassemia, pancreatitis, and fatty liver disease [6]. In our case presentation, FES was suspected to be linked to the sickle cell (SC) trait, which, to the best of our knowledge, has never been reported before.

In SCD patients experiencing vaso-occlusive crises (VOC), BMN is often found upon examination of the bone marrow. While a bone marrow biopsy can provide support for the diagnosis of FES, it cannot definitively rule it out. It is worth noting that typical cases of FES may not exhibit extensive BMN on biopsy, which can be attributed to the timing of the sample collection, as bone marrow tends to rapidly recover after an acute event [5].

Diagnosing FES can be challenging as clinical symptoms, laboratory findings, and imaging results associated with the condition are typically nonspecific. Currently, there are no standardized criteria for defining FES. Two diagnostic criteria, Gurd's criteria [7,8] and Schonfeld's criteria [9], have been proposed, among others. These criteria primarily rely on a high level of suspicion, the clinical presentation, basic diagnostic tests, and the absence of other potential causes. They are commonly used as diagnostic guidelines for traumatic FES.

When FES affects the brain, it can lead to scattered strokes, either hemorrhagic or ischemic, resulting in various manifestations such as headaches, altered mental states, and, less commonly, coma and brain death [10]. Cerebral fat embolism (CFE) significantly contributes to morbidity and mortality associated with FES, making central nervous system (CNS) involvement a primary concern during FES episodes [11].

While CFE and cerebral infarctions have been reported in individuals with SCD, our case is unique as this phenomenon has never been described in a patient with the sickle cell trait. Our presentation follows an extensive diagnostic workup to exclude other potential causes and is presumed to have been triggered by a parvovirus infection.

## Case presentation

A 54-year-old male with a past medical history of coronary artery disease (CAD) status post-coronary artery bypass grafting (CABG), diabetes mellitus (DM), atrial fibrillation, and a recent left eye surgery (using iodine-based drops for persistent retinal hemorrhage) who presented to our facility with a chief complaint of confusion and visual hallucinations. Vitals on presentation were remarkable for fever and tachycardia. An initial physical exam showed encephalopathy that had been progressively worsening over the past two weeks and bilateral lower extremity weakness. His mental status continued to deteriorate and resulted in a coma state requiring intubation for airway protection. No history or clinical signs of trauma were reported. 

**Figure 1:**
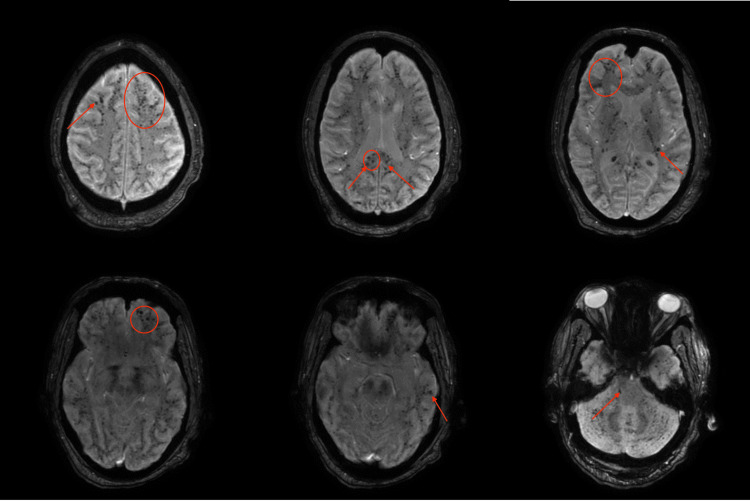
Axial GRE sequence of MRI brain that is showing diffuse multiple hemorrhages related to FES in our patient (some lesions outlined by arrows and circles)

CT head without contrast showed no acute findings with nonspecific leukoaraiosis. Given the neurological symptoms and fever on presentation, a lumbar puncture was done to rule out infection. Cerebrospinal fluid (CSF) showed lymphocytic pleocytosis and moderately elevated protein (Glucose 169, protein 67, WBC 40 (lymphocytic predominance), RBC 26); however, cultures and meningitis and encephalitis panel were negative for any infectious etiologies. As part of the workup, an MRI brain without contrast showed diffuse tiny foci of microhemorrhages throughout the brain that were evident on the gradient echo (GRE) sequence (Figure 1). After the earlier workup was done, our differentials were diffuse axonal injury, thrombocytopenic purpura (TTP), neurodegenerative disorders, and vasculitis.

Given thrombocytopenia and renal impairment, TTP or vasculitis was suspected, and the patient was treated initially with steroids and plasma exchange. However, extensive workup was negative for vasculitis, including angiography along with negative disintegrin and metalloproteinase with a thrombospondin type 1 motif, member 13 (ADAMTS13), that ruled out TTP. Additionally, a workup for infectious etiologies was negative.

Given the lack of a clear etiology after an extensive initial workup evaluating the different causes of thrombocytopenia and anemia, which included blood counts, peripheral smear, and reticulocyte counts, we proceeded with a bone marrow biopsy, which showed diffuse BMN. This finding yielded further workup that came back positive for SC trait and positive parvovirus IgG as well.

Tables 1-2 summarize the lab and imaging findings.

**Table 1:** Summary of the laboratory findings

BIOCHEMICAL FINDINGS		
CBC	Thrombocytopenia and anemia normal WBC and reticulocytes	
PERIPHERAL SMEAR	Negative	
BLOOD CULTURE	Negative	
PARVOVIRUS IGG	Positive	
URINALYSIS AND CULTURE	Negative	
LP	WBC: 40; RBC: 26; Protein: 67; Glucose: 169; meningitis and encephalitis panel: negative	
ADAMST3	Negative	
BM BIOPSY	Diffuse BMN	
HEMOGLOBIN ELECTROPHORESIS	SC trait (Hg AS)	

**Table 2:** Summary of imaging results

RADIOLOGICAL FINDINGS	
CT HEAD	No acute findings with nonspecific leukoaraiosis
MRI BRAIN	Diffuse tiny foci of microhemorrhages on GRE
COVENTINAL ANGIOFRAPHY	Negative for vasculitis features

Putting all the results together, this was considered a case of acute FES that was reported before with patients with SCD and rarely SC trait during an acute crisis, especially with parvovirus coinfection [12]. After prolonged hospitalization, our patient underwent tracheostomy and feeding tube placement, and he was transferred to a nursing home with minimal improvement in his mental status or general exam.

## Discussion

That being said, in more detailed definitions previously published, FES has been described as either the involvement of multiple or single organs confirmed histologically by the presence of fat and/or necrotic marrow emboli, the development of acute respiratory distress and neurological symptoms, or multiorgan failure with evidence of bone marrow necrosis (either through pathological evidence or laboratory findings). This expanded definition may be more suitable [12]. FES can occur after traumatic injuries, orthopedic surgeries, or other medical conditions.

One of the rare causes of FES is SCD. The earliest documented case of FES in an SCD patient dates back to 1941. This patient had experienced multiple vaso-occlusive episodes over six years. However, in the last episode, he presented with cerebral symptoms [13] and was found to have what we now call CFE. After this report, more cases were reported in the literature. 

SCD-causing FES is usually connected with a VOC. The pathogenesis of FES in the setting of SCD has been attributed to two main mechanisms: a mechanical one that happens because of bone marrow necrosis and a metabolic theory that suggests that emboli arise because of some biochemical change initiated by tissue damage, such as increased C-reactive protein [14-16]. Notably, the current diagnostic criteria for FES are designed for traumatic FES and do not apply to atraumatic or SCD-related FES.

One potential risk factor suggested for FES in the context of VOC is Parvovirus coinfection. A review of the literature identified 41 cases of FES/BMN in SCD patients reported after 1986, with only 10 cases having positive confirmatory tests for parvovirus coinfection. Four cases had documented negative testing, while the remaining cases did not mention their parvovirus testing status [17]. The exact connection between parvovirus coinfection and FES in SCD patients remains unknown, and further exploration and investigation are needed. If confirmed as a risk factor, this could enhance our understanding of the pathophysiology of FES in SCD patients.

It has been observed that the most severe forms of FES, often with fatal outcomes, are associated with the hemoglobin SC variant of SCD (referred to as the non-SS genotype). However, the reason behind this observation is yet to be discovered [18].

Most of the FES cases reported in the literature were associated with SCD. Our case presentation was assumed as a rare FES case in the setting of SC traits (which have never been reported before), particularly in the presence of parvovirus coinfection during an acute crisis. FES can be missed given the similar presentation to other etiologies like sepsis and vasculitis, which are much more common [5,19]. Diagnosing FES early is vital, as this potentially will alter the course and prognosis of the cases if earlier therapies are applied [20]. Despite its rarity and the requirement for a high index of suspicion for diagnosis, we believe it is essential to develop or revisit diagnostic criteria to help with early recognition and treatment of FES.

## Conclusions

Our case was challenging given the lack of clear diagnostic criteria for presumed FES and the lack of clear etiology despite the extensive workup, as mentioned earlier. Hence, we assumed that this is a rare presentation of an FES in the setting of an SC trait in the context of parvovirus infection.

Blood transfusion or exchange transfusion, along with supportive care, has represented the only treatment available for FES in the context of SCD until today. In this case demonstration, we observed an uncommon case of progressive encephalopathy resulting from FES in a patient with the SC trait.
